# High-pass filter settings and the role and mechanism of discrete ventricular electrograms in left bundle branch pacing

**DOI:** 10.3389/fcvm.2022.1059172

**Published:** 2023-01-11

**Authors:** Jiabo Shen, Longfu Jiang, Hao Wu, Hengdong Li, Lu Zhang, Jinyan Zhong, Shanshan Zhuo, Lifang Pan

**Affiliations:** ^1^Department of Cardiology, Hwa Mei Hospital, University of Chinese Academy of Sciences, Ningbo, Zhejiang, China; ^2^Department of Global Health, Ningbo Institute of Life and Health Industry, University of Chinese Academy of Sciences, Ningbo, Zhejiang, China

**Keywords:** left bundle branch pacing (LBBP), conduction system pacing, discrete electrogram, isoelectric interval, high-pass filter settings

## Abstract

**Objective:**

The characteristics of discrete intracardiac electrogram (EGM) in selective left bundle branch (SLBB) pacing (SLBBP) have not been described in detail previously. This study aimed to examine the effect of different high-pass filter (HPF) settings on discrete local ventricular components in an intracardiac EGM and to analyze its possible mechanisms.

**Methods:**

This study included 144 patients with indications of permanent cardiac pacing. EGMs were collected at four different HPF settings (30, 60, 100, and 200 Hz) with a low-pass filter at 500 Hz, and their possible mechanisms were analyzed.

**Results:**

LBBP was successfully achieved in 91.0% (131/144) of patients. SLBBP was achieved in 123 patients. The occurrence rates of discrete local ventricular EGM were 16.7, 33.3, 72.9, and 85.4% for HPF settings of 30, 60, 100, and 200 Hz, respectively. The analysis of discrete EGM detection showed significant differences between the different HPF settings. By using the discrete local ventricular component and isoelectric interval as the SLBB capture golden standard, the results of EGMs revealed that the 30 Hz HPF has a sensitivity of 19% and specificity of 100%. The 60 Hz HPF had a sensitivity of 39% and a specificity of 100%. The 100 Hz HPF had a sensitivity of 85% and a specificity of 100%. The 200 Hz HPF had a sensitivity of 100% and specificity of 100%.

**Conclusion:**

An optimal HPF setting of 200 Hz is recommended for discrete local ventricular EGM detection. A discrete local ventricular EGM should exhibit an isoelectric interval. A steep deflection and high-frequency ventricular EGM morphology nearly identify an intrinsic EGM morphology.

## Highlights

- Left bundle branch (LBB) pacing (LBBP) is a novel native conduction system pacing strategy.
- Identifying discrete local ventricular electrogram (EGM) is crucial for accurately diagnosing selective LBB capture.
- Identifying discrete local ventricular EGM is a challenging task.
- This study aimed to evaluate the diagnostic accuracy of discrete local ventricular EGMs by adjusting high-pass filters with different settings in LBBP.
- The morphology of the discrete local ventricular EGMs was retrospectively observed and analyzed to explore the possible mechanisms of their formation.
- Different high-pass settings do not affect the identification of Purkinje potential.

## Introduction

Left bundle branch (LBB) pacing (LBBP) is a novel native conduction system pacing strategy (1). Changes in the intracardiac ventricular electrograms (EGMs) are usually assessed during LBBP implantation via an electrophysiological recording system (EPS) (2). A discrete local ventricular EGM and an isoelectric interval have been previously used as criteria to confirm the selective LBB (SLBB) capture, which suggests that only the conduction system was captured, and the myocardium was lost. Therefore, identifying discrete local ventricular EGM is crucial for accurately diagnosing SLBB capture (3). Filtered unipolar electrograms were obtained in previous studies, usually with settings of 30 and 100/300/500 Hz (4–6), for LBBP. However, filtering could remarkably change the morphology of the ventricular EGM, introducing the possibility of errors in the evaluation of electrograms. When the high-pass filter (HPF) of the LBB lead channel is set to 30 Hz and clipping is set at 3 cm, the entire ventricular endocardial signal may not be observed owing to the large amplitude in the EGM. Therefore, identifying discrete local ventricular EGMs is a challenging task. We hypothesized that an HPF other than 30 Hz improved the detection of discrete local ventricular EGM. Therefore, in the present study, we aimed to evaluate the accuracy of discrete local ventricular EGM in diagnosing selective LBBP (SLBBP) in different HPF settings and analyze its possible mechanisms.

## Materials and methods

### Patient population and definition of LBB capture

This retrospective observational study enrolled consecutive patients who underwent successful permanent pacemaker implantation. LBBP uses John Jiang’s connecting cable (Xinwell Medical Technology Co., Ltd., Ningbo, Zhejiang, China) for the continuous pacing and recording technique, and the procedure has been described by us elsewhere (7–10). The study protocol was approved by the Ethics Committee of the Hwa Mei Hospital, Ningbo, China. Written informed consent was obtained from all patients.

Successful LBBP is defined as follows. LBB capture is characterized by paced QRS morphology of the right bundle branch block (RBBB) pattern and all of the following criteria: (1) differential pacing at 8 and 2 V, producing the shortest and constant V6 R-wave peak time and (2) demonstration of left ventricular septal (LVS) to non-selective LBB capture transition during constant output while advancing the lead and non-selective to SLBB capture during unipolar pacing threshold assessment (4, 11). The discrete local ventricular component and isoelectric interval with decrementing output during the EGM recording were used as the golden standard of the SLBB capture (3, 4, 12, 13).

### Data recording and analysis

The baseline patient characteristics and indications for pacing were documented. Twelve-lead electrocardiogram (ECG) and EGM from the pacing lead were continuously recorded with an EPS (EP-Workmate, Abbott Laboratories, Chicago, IL, USA). For each patient, high- and low-pass filter (LPF) settings of 200 and 500 Hz were performed during the live case (9). The differences in discrete local ventricular EGM morphologies were collected and analyzed offline using four different HPF settings (30, 60, 100, and 200 Hz), and the LPF was set at 500 Hz. To ensure high precision, the analysis of discrete local ventricular EGM morphology was performed using endocardial channel recording, digital calipers, fast sweep speed (200 mm/s), and appropriate signal augmentation. The clipping was set at 3 cm, and the amplitude was set at 0.5 mV/cm.

The characteristics of the various transitions in discrete local ventricular EGM morphology were analyzed retrospectively after the procedure. All EGM morphologies were independently analyzed by two medical practitioners who were highly experienced in EGM interpretation. When (1) the isoelectric interval, (2) the paced initial steep deflection, and (3) high-frequency local ventricular EGM nearly identical to the intrinsic ventricular EGM could be observed independently by both doctors at different HPF settings, the observation was marked as a discrete local ventricular EGM (patients with left bundle branch block (LBBB) meeting criteria 1 and 2 because intrinsic LBB conduction cannot be observed). The EGM readers were blinded to the study’s purpose. In the absence of concordance between the two readers, a third cardiologist practitioner adjudicated the results.

### Statistical analysis

All continuous data are presented as mean ± standard deviation (SD). Categorical data were presented as numbers and percentages. We used Student’s *t*-test to compare continuous variables. To evaluate the diagnostic accuracy of detecting discrete local ventricular EGM, the sensitivity, specificity, positive predictive value (PPV), and negative predictive value (NPV) of different HPF settings were calculated. A *p*-value < 0.05 was considered statistically significant. The statistical software IBM SPSS Statistics for Windows (version 26.0, IBM Corp, Armonk, NY, USA) was used for analysis.

## Results

From April 2021 to September 2022, data for 144 patients who underwent pacemaker implantation were consecutively and retrospectively collected from a single institution (Hwa Mei Hospital, University of Chinese Academy of Sciences). Their mean age was 73.9 ± 9.2 years, and 63/144 (43.8%) were females. Clinical and procedure-related characteristics of the study population are shown in Table 1. Successful LBBP with evidence of LBB system capture was achieved in 131 patients (91.0%). SLBBP was achieved in 123 patients (85.4%) during the threshold testing. Eight patients were confirmed as having non-selective LBBP (NSLBBP). In thirteen patients, LBBP failed because of the inability to capture the LBB. These patients eventually underwent LVS pacing. The pacing indications in the 131 patients who achieved LBBP were sick sinus syndrome in 44 (33.6%), atrioventricular block in 84 (64.1%), atrial fibrillation with bradycardia in 7 (5.3%), and heart failure in 5 (3.8%). LBB potential (Po_*LBB*_) was recorded in 94 (71.9%) of 131 patients.

**TABLE 1:** Baseline characteristics, pacing indications, and baseline echocardiography and ECG data of patients who underwent attempts at LBBP.

	LBBP (*n* = 131)	LVSP (*n* = 13)	*p*
Age (years)	73.5 ± 9.1	77.3 ± 9.9	0.51
Male	77 (60.2)	7(53.4)	
**Pacing indication (*n*)**			
Atrioventricular block	84 (64.1)	9 (69.2)	
Sick sinus syndrome	44 (33.6)	2 (15.4)	
Atrial fibrillation with bradycardia	7 (5.3)	2 (15.4)	
Heart failure	5 (3.8)	0(0)	
**Comorbidities (*n*)**			
Hypertension	77 (58.8)	10 (76.9)	
Diabetes mellitus	35 (26.7)	3 (32.1)	
Cardiomyopathy	10 (7.6)	0(0)	
Coronary heart disease	23 (17.6)	3 (23.1)	
Atrial fibrillation	37 (28.2)	5 (38.5)	
LVEF (%)	63.6 ± 10.2	65.0 ± 6.4	0.46
LVDD (mm)	49.9 ± 7.2	50.2 ± 3.6	0.07
**QRS morphology (*n*)**			
Narrow QRS	95 (72.5)	8(61.5)	
RBBB	21 (23.9)	0(0)	
LBBB	14 (15.9)	0(0)	
NIVCD	2 (2.3)	1(14.3)	
**Procedure-related parameters**			
LBB potential observed (*n*)	94 (71.9%)	0(0)	
Threshold (V/0.5 ms)	0.61 ± 0.41	0.61 ± 0.33	0.99
R-wave amplitude (mV)	14.7 ± 6.7	9.9 ± 4.0	0.05
Impedance (Ω)	733.7 ± 137.6	730.7 ± 120.1	0.69
Lead depth (mm)	14.8 ± 2.6	14.6 ± 1.5	0.07

Patients who underwent LVSP were those in whom LBBP failed. LVEF, left ventricular ejection fraction; LVDD, left ventricular end-diastolic dimension; RBBB, right bundle branch block; LBBB, left bundle branch block; NIVCD, non-specific intraventricular conduction disturbance; LBB, left bundle branch; LBBP, left bundle branch pacing; LVSP, left ventricular septal pacing. Continuous data were presented as mean ± standard deviation.

*p* < 0.05 was considered statistically significant.

The performance of the discrete local ventricular EGM detection with different HPF is shown in Figures 3–5. The discrete local ventricular EGM occurrence rates for different EGM setup channels were compared. The results of the discrete local ventricular EGM detection are summarized in Tables 2, 3. The occurrence rates of discrete local ventricular EGM were 16.7% (24/144), 33.3% (48/144), 72.9% (105/144), and 85.4% 123/144) for HPF settings of 30, 60, 100, and 200 Hz, respectively (Table 2). The analysis of discrete ECG detection showed significant differences between the different HPF settings (Table 3). Using the discrete local ventricular component and isoelectric interval as the SLBB capture gold standard, the results of EGMs indicated that the 30 Hz HPF had a sensitivity of 19% and specificity of 100%. Furthermore, the PPV was 100 and 17%, respectively. The 60 Hz HPF had a sensitivity of 39% and specificity of 100% for SLBB capture, with a PPV of 100% and NPV of 21%. The 100 Hz HPF had a sensitivity of 85% and specificity of 100%, with a PPV of 100% and NPV of 53%. The 200 Hz HPF had a sensitivity of 100% and specificity of 100% for SLBB capture, with a PPV of 100% and NPV of 100%.

**FIGURE 1:**
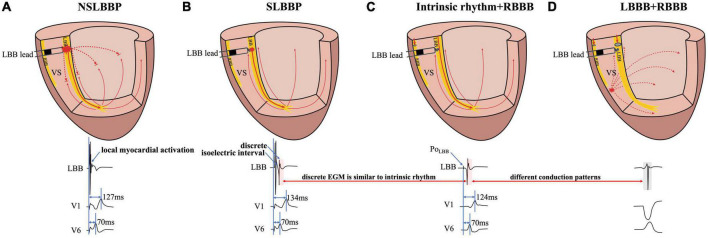
Schematic diagram of different conduction patterns in a patient with RBBB and intermittent LBBB. **(A)** NSLBBP captures both the local myocardium and LBB without the presence of isoelectric interval and discrete local ventricular EGM. **(B)** In SLBBP, pacing electrical stimulation is conducted through the conduction system to the apex and propagates to the base of the interventricular septum. Isoelectric interval and discrete local ventricular EGM were observed. **(C)** Intrinsic conduction antegrade to the apex and propagates to the base. Morphology of intrinsic and paced ventricular EGMs was nearly identical (red rectangle). **(D)** The EGM morphology of LBBP is similar to the native rhythm but different from the EGM morphology of LBBB (red and gray rectangle). NSLBBP, non-selective left bundle branch pacing; SLBBP, selective left bundle branch pacing; EGM, intracardiac electrogram; LBB, left bundle branch; LBBB, left bundle branch block; RBBB, right bundle branch block; VS, ventricular septum.

**FIGURE 2:**
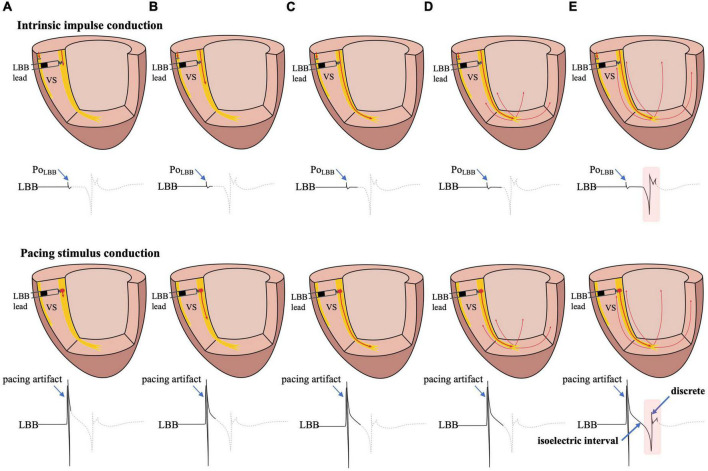
The possible mechanism of the formation of the isoelectric interval and discrete electrogram. **(A)** Intrinsic impulse or pacing stimulus in the LBB is sensed by the tip lead. **(B–D)** The formation process of the isoelectric interval. **(E)** Intrinsic impulse or pacing stimulus reaches the distal end of the His-Purkinje system, excites the apical myocardium, propagates into the basal myocardium, and is then sensed by the tip lead, manifesting as an isoelectric interval and discrete electrogram. Abbreviations as in Figure 1.

**FIGURE 3:**
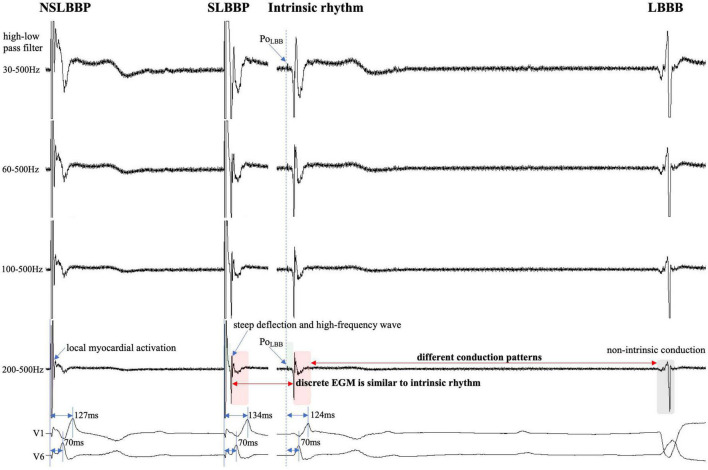
Discrete local ventricular EGM morphology resembles ventricular EGM morphology of intrinsic rhythm (red rectangle). The EGM morphology of LBBP is similar to the native rhythm but is completely different from the EGM morphology of LBBB (red and gray rectangle). Po_LBB_ can be observed at different high-pass filter settings (blue dashed line). Isoelectric interval does not appear during local myocardial activation (purple rectangle). Isoelectric interval is affected by pacing artifacts (green rectangle). Abbreviations as in Figure 1.

**FIGURE 4:**
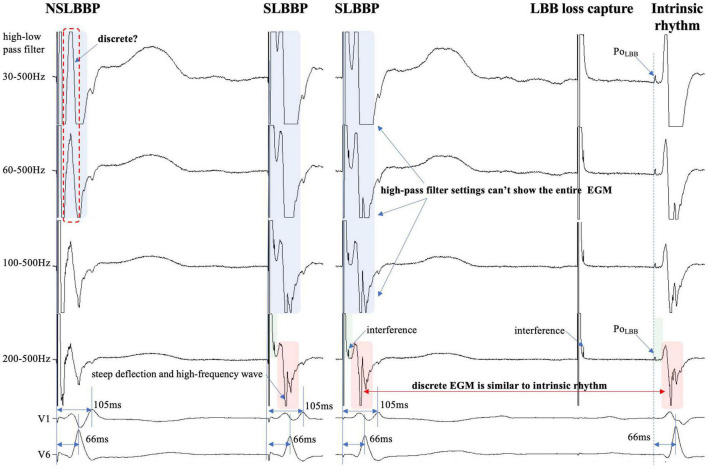
Discrete ventricular components cannot be accurately identified because the entire EGM morphology cannot be displayed (blue rectangle). Steep deflection and high-frequency discrete local ventricular EGM morphology are similar to ventricular EGM morphology of intrinsic rhythm (red rectangle). Isoelectric interval is affected by pacing artifacts (green rectangle). Po_LBB_ can be observed at different high-pass filter settings (blue dashed line). Abbreviations as in Figure 1.

**FIGURE 5:**
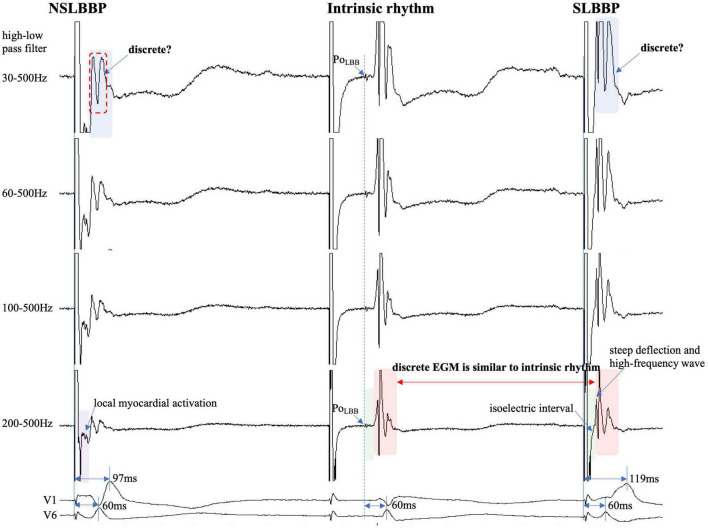
Discrete ventricular components cannot be accurately identified because the entire EGM morphology cannot be visualized (blue rectangle). Discrete local ventricular EGM morphology resembles ventricular EGM morphology of intrinsic rhythm (red rectangle). The isoelectric interval of paced rhythm and native rhythm (green rectangle). Changes in high-pass filter settings do not affect the identification of Po_LBB_ (blue dashed line). Isoelectric interval does not appear during local myocardial activation (purple rectangle). Abbreviations as in Figure 1.

**TABLE 2:** Detection of discrete local ventricular EGM at different high-pass filter settings.

	30 Hz	60 Hz	100 Hz	200 Hz
Presence of discrete local ventricular EGM	24 (16.7)	48 (33.3)	105 (72.9)	123 (85.4)
Absence of discrete local ventricular EGM	120 (83.3)	96 (67.7)	39 (27.1)	21(14.6)

Data are presented as numbers (%). EGM, intracardiac electrogram.

**TABLE 3:** Results and diagnostic accuracy of different high-pass filter for detecting discrete local ventricular EGM.

	30 Hz	60 Hz	100 Hz	200 Hz
Sensitivity % (95% CI)	0.19 (0.13–0.27)	0.39 (0.30–0.48)	0.85 (0.77–0.91)	1.00 (0.96–1.00)
Specificity % (95% CI)	1.00 (0.80–1.00)	1.00 (0.80–1.00)	1.00 (0.80–1.00)	1.00 (0.80–1.00)
PPV % (95% CI)	1.00 (0.82–1.00)	1.00 (0.90–1.00)	1.00 (0.95–1.00)	1.00 (0.96–1.00)
NPV % (95% CI)	0.17 (0.11–0.25)	0.21 (0.14–0.31)	0.53 (0.37–0.69)	1.00 (0.80–1.00)

95% CI, 95% Confidence interval; EGM, intracardiac electrogram; NPV, negative predictive value; PPV, positive predictive value.

## Discussion

Left bundle branch pacing includes NSLBBP and SLBBP. In NSLBBP, both the LBB and the local ventricular myocardium are directly captured by the pacing stimulus, in parallel and not in sequence and therefore the paced ventricular EGM morphology is not identical to the local native ventricular EGM morphology (Figure 1A). SLBBP was defined as only capturing the LBB with a typical RBBB morphology as well as a discrete isoelectric component between the pacing stimulus and the onset of discrete and identical local ventricular activation due to local myocardium not being directly captured (Figure 1B). A discrete local ventricular EGM is a characteristic of an SLBBP (2). It appears as a current deflection wave with a short isoelectric interval and large amplitude with an HPF setting of 30 Hz.

The isoelectric interval is often observed between the pacing artifact and the paced discrete ventricular component. This phenomenon includes the true isoelectric interval (time required for immediate peri-electrode tissue excitation or a local response) and local conduction time (time required for propagated excitation to recruit sufficient local myocardial tissue to produce the ventricular EGM) (14). Typically, the isoelectric interval is short (<30 ms) (4). An increased isoelectric interval may result from nonhomogeneous excitation propagation from the stimulation site and conduction delay in the His–Purkinje system (14). A previous study positioned a linear multielectrode catheter along the left ventricular septum to record intracardiac signals from the base to the apex to assess left ventricular activation sequences (15). According to the “V”-shaped conduction pattern observed in this study, the mechanism underlying the formation of the isoelectric interval may be associated with the propagation of impulse or pacing stimulus through the conduction system, reaching the distal part of the His–Purkinje system to excite the apical myocardium, and subsequent propagation to the basal myocardium in the interventricular septum by the electrode sensing (Figure 2). In non-LBBB patients, the discrete local ventricular EGM was nearly identical to the native ventricular EGM morphology (Figures 1–5, red rectangle). Intrinsic and paced ventricular EGMs were nearly identical in patients with intermittent LBBB with native anterograde conduction (Figure 3, red rectangle). Therefore, we speculate that in patients with complete LBBB, although the native rhythm (intrinsic conduction) cannot be observed, the EGM morphology of the intrinsic LBB conduction should be consistent with the EGM morphology of LBB pacing. Additionally, we observed inconsistent ventricular EGM morphology on the tip lead due to different LBBB and intrinsic conduction pathways (Figure 3, gray rectangle).

In previous studies, high- and LPF settings of 30 and 500 Hz were set to record the EGM. Clinicians usually employ a 30 Hz HPF to record Po_LBB_ (5). However, with this HPF, discrete local ventricular EGMs may be missed easily because the clipping level limits the display range and does not allow the user to view large-amplitude endocardial signals (Figures 4, 5, blue rectangle). Additionally, in some patients, demonstration of the isoelectric interval and discrete local electrogram may be challenging due to short stimulus to ventricular intervals, effects of stimulus artifact, and far-field recording by the pacing lead (Figures 3–5, green rectangle) (16). In contrast, pacing artifacts and separation of ventricular components can be observed in some cases, which are easily confused with true discrete local ventricular EGM (Figures 4, 5, red dashed frame). However, these observations do not represent the SLBB capture. Based on the observations in our study, the paced initial steeply deflected ventricular EGM morphology should be nearly identical to the intrinsic ventricular EGM morphology with an isoelectric interval to be considered SLBBP (Figures 1–5, red rectangle).

The HPF is designed to eliminate unwanted lower frequencies by allowing frequencies higher than the filter settings to pass. The higher the frequency, the lower the baseline wander. The LPF passes frequencies lower than the cutoff frequency and attenuates higher frequencies. The lower the frequency, the lower is the baseline noise. The change in the signal produced by filtering depends on the frequency of the unfiltered signal. Variations in the HPF produced marked changes in electrogram morphology, introducing the possibility of inaccurately assessing discrete local ventricular EGM (Figures 4, 5, blue rectangle). Accurate interpretation of discrete local ventricular EGMs highly depends on the magnitude of the ventricular component. An excessive amplitude affects the identification of a discrete local ventricular EGM. Therefore, with clipping set to 3 cm and amplitude set to 0.5 mV/cm, we attempted to show the intact and entire ventricular EGM more clearly by adjusting the HPF setting to confirm SLBB capture by identifying discrete local ventricular EGM and isoelectric interval.

To the best of our knowledge, no previous study has identified a discrete local ventricular EGM by adjusting the band-pass filter with different setup conditions in LBBP. One purpose of this study was to define the impact of different HPF settings on the accuracy of discrete electrogram identification. These parameters were compared for different HPF values of 30, 60, 100, and 200 Hz. The unipolar electrogram signal morphology was subsequently analyzed. Our research suggested that the occurrence rates of discrete local ventricular EGM were 16.7, 33.3, 72.9, and 85.4% for HPF settings of 30, 60, 100, and 200 Hz, respectively. Although some discrete local ventricular EGM can be observed at 30 Hz HPF, the sensitivity is low (sensitivity, 19%; specificity, 100%) and it is difficult to accurately identify all discrete local ventricular EGM. However, the 200 Hz HPF had a sensitivity of 100% and specificity of 100% for SLBB capture. When 30 Hz HPF identification of discrete local ventricular EGM was difficult, the 200Hz setting can accurately identify discrete local ventricular EGM (Figures 4, 5). This means that this HPF setup can identify all discrete local ventricular EGM. For other HPF such as 300 Hz, we also tried and found that the sensitivity was still 100% but would affect the identification of the Po_LBB_. The result suggests that an optimal HPF setting of 200 Hz is recommended for detecting discrete local ventricular EGM. We also tried adjusting the LPF to observe the discrete local ventricular EGM, but found that these settings did not increase the identification accuracy of the discrete local ventricular EGM. Moreover, a discrete Purkinje potential precedes the onset of local ventricular EGM. Filtered unipolar electrograms were obtained at 30 Hz and 500 Hz to record the Po_LBB_ (6). An LPF was used to eliminate the noise. In our study, the LPF was 500 Hz, although the HPF settings differed. This indicates that such an HPF setting does not affect the identification of the Po_LBB_ (Figures 3–5, blue dashed line).

## Study limitations

This study has several limitations. This retrospective study was performed at a single center and included a relatively small number of patients. Although the intrinsic and paced EGMs were also nearly identical in patients with intermittent LBBB, there is still a lack of evidence suggesting that paced discrete local ventricular EGM is nearly identified as native EGM in patients with complete LBBB. We used only one particular manufacturer EPS. It is possible that the ability to detect discrete local ventricular EGM might differ depending on the EPS used because of differences in signal processing algorithms. Therefore, it is unknown whether the results of this study can be extended to other patient groups or different EPSs. To address these issues, a larger study including different patient groups and EPSs is needed. Randomized controlled and prospective trials are needed to confirm the findings of this study and to provide guidance to clinicians. Outcome data in terms of persistence of the sensed findings, ventricular function, or quality of life-based are lacking.

## Conclusion

We demonstrated that an HPF setting of 30 Hz, routinely used in clinical practice, cannot reliably meet the clinical requirements of discrete local ventricular EGM detection. Our results suggest that clinicians can adjust HPF appropriately to improve discrete local ventricular EGM diagnosis, and a 200 Hz filter may be a desirable choice. A discrete local ventricular EGM should show an isoelectric interval, and a steep deflection and high-frequency ventricular EGM morphology nearly identify an intrinsic EGM morphology.

## Data availability statement

The raw data supporting the conclusions of this article will be made available by the authors, without undue reservation.

## Ethics statement

The study protocol was approved by the Ethics Committee of the Hwa Mei Hospital, Ningbo, China. The patients/participants provided their written informed consent to participate in this study. Written informed consent was obtained from the individual(s) for the publication of any potentially identifiable images or data included in this article.

## Author contributions

JS and LJ conceived and designed the experiment. HW and LZ analyzed the data. JZ and SZ performed the statistical analysis. JS and LP wrote the manuscript. HL and LJ revised the manuscript. All authors contributed to the article and approved the submitted version.
